# Prevalence of erectile dysfunction and associated factors among diabetic men attending diabetic clinic at Muhimbili National Hospital in Dar-es-Salaam, Tanzania

**DOI:** 10.11604/pamj.2014.17.227.2695

**Published:** 2014-03-26

**Authors:** Reuben Kato Mutagaywa, Janeth Lutale, Muhsin Aboud, Benjamin Anathory Kamala

**Affiliations:** 1 Muhimbili University College of Health and Allied Sciences Post Office Box 65000, Dar es Salaam, Tanzania; 2 Muhimbili Orthopaedic Institute, Post Office Box 65474, Dar-es-Salaam, Tanzania; 3 PeerCorps Trust Fund, 352/64 Makunganya Street, Co-Architecture Building, 4th Floor, Post Office Box 22499, DSM, Tanzania

**Keywords:** Diabetes mellitus, erectile dysfunction, sexual domains, peripheral vascular disease, monofilament score, peripheral neuropathy

## Abstract

**Introduction:**

There has been an increase in the prevalence of erectile dysfunction (ED) in the general population especially among Diabetic patients. This seems to be neglected problem in low-income countries. This study aims at establishing the prevalence of ED and associated risk factors in diabetic patients attended at Diabetic Clinic at Muhimbili National Hospital.

**Methods:**

A cross-sectional hospital based study was conducted among 312 diabetic patients attending diabetic clinic at Muhimbili National Hospital between May and December 2011.

**Results:**

More than half (55.1%) of the patients were found to have some form of ED (12.8% had mild dysfunction, 11.5% moderate and 27.9% severe dysfunction). The severity of ED was correlated with increased age. Multivariate logistic regression revealed that ED was significantly predicted by old age (odds ratio (OR) = 7.1, 95% CI 1.2-40.7), evidence of peripheral neuropathy (OR) =5.9, 95% CI 1.6-21.3), and evidence of peripheral vascular disease (OR =2.5, 95% CI 1.2-5.3). Also longer duration of DM was marginally associated with ED (p=0.056). Patients with ED were also more likely to suffer other sexual domains (p<0.001). No lifestyle factor was associated with ED.

**Conclusion:**

The prevalence of ED is high among DM patients. Interventions aimed at prevention, early diagnosis and detection of DM and its complications, and adherence to treatment to prevent complications should be implemented. Further studies should emphasize on temporal variation to show true causality of DM on erectile dysfunction.

## Introduction

Erectile dysfunction (ED) defined as inability to achieve and maintain an erection sufficient to permit satisfactory sexual intercourse is documented to be a major problem especially among diabetic patients [1].

There are numerous causes of ED generally falling into two categories, organic or psychogenic. The organic causes can be subdivided into five categories: vascular, traumatic/post surgical, neurological, endocrine-induced, and drug-induced. Reported psychogenic causes include depression, performance anxiety, and relationship problems [2].

A variety of chronic illnesses such as diabetes mellitus DM), cardiovascular disease and depression are associated with higher rates of ED [3] where DM appears to be a major determinant [4]. In study that was done in Massachusetts, United States of America showed that diabetic men are 3 times as likely to develop ED as non-diabetic men [5]. Also increased duration of diabetes was shown to increases both the prevalence rate and severity of ED [6]. ED can occur early in the course of the disease and it can occasionally be the presenting symptom [7]. Some documented reports gave out the main risk factors of ED in people with diabetes as neuropathy, vascular insufficiency, poor glycemic control, hypertension, low testosterone levels, and lifestyle factors such as smoking, alcohol and inactivity. In addition the prevalence of ED greatly increases with age [8–11].

A wide range of prevalence rates of ED among diabetic men has been reported in various studies. These studies [6, 12–15] suggest that its prevalence in men with diabetes ranges from 35–90% versus 26% in general population. Variation in prevalence is mainly due to differences on definitions used, differences in the populations studied, study design, classification, and the diagnostic tools- especially for studies conducted in the past. Introduction of validated questionnaire (the IIEF) minimized this variation [2].

Many male sexual function profiles and ED questionnaires have been developed. The International Index of Erectile Function Questionnaire (IIEF) is the most reliable measure of ED [3, 16]. It has 15 questions and the total score is obtained by the sum of the individual scores of each question. It addresses and quantifies five domains, namely: erection function, orgasmic (ejaculation) function, sexual desire (libido), intercourse satisfaction (ability to sustain intercourse), and overall satisfaction/premature ejaculation.

The current study aimed at determining the prevalence of erectile dysfunction and associated factors among diabetic men attending the diabetic clinic at Muhimbili National Hospital (MNH), Dar-es-Salaam.

Specifically the study wanted to answer the following questions: to determine the prevalence of erectile dysfunction among male diabetic patients attending diabetic clinic at MNH, Dar-es-Salaam, to determine the severity of erectile dysfunction among male diabetic patients attending diabetic clinic at MNH, Dar-es-Salaam, to determine factors influencing erectile dysfunction among male diabetic patients attending diabetic clinic at MNH, Dar-es-Salaam, and to describe the association between erectile dysfunction and other sexual function domains among male diabetic patients attending diabetic clinic at MNH, Dar es Salaam.

## Methods

This was a cross section hospital based prospective study, which was conducted at MNH for the period of 8 month from May to December 2011. The study population included all adult (≥ 18 years) diabetic men attending diabetic clinic at MNH with duration of diabetic ≥ 1 year. We excluded very sick patients who were defined as: Patients with unstable vital signs/mental status e.g. in diabetic ketoacidosis or hyperosmolar hyperglycemic state, confused, or in septicemia as in infected diabetic foot.

During recruitment, the principal investigator (PI) filled in a standardized structured questionnaire for each patient included in the study. The questionnaire included socio-demographic and clinical aspects data, to assess the different sexual domains the International Index of Erectile Function (IIEF) Questionnaire was used. The IIEF was translated into Kiswahili language and the author asked all the 15 questions.

Measures included Socio-demographic data and Medical history regarding duration of diabetes, type of diabetes, medications taken for diabetes; presence of co morbid conditions was obtained. Moreover, anthropometric measurements were recorded and blood for laboratory tests was taken.

Height (in meters) was taken using a commercially available stadiometer (AXIOM, AX - 120) and weight (in kilograms) was measured using a standard weighing. The body mass index (BMI) was derived by dividing the weight (kg) by the square of the height (m) and classification of obesity was done according to World Health Organization [17] criteria.

Blood pressure measurement using standard well-calibrated mercury sphygmomanometer based was taken. Hypertension was defined as SBP ≥ 140mmHg and/or DBP ≥ 90mmHg [18] or known hypertensive on treatment.

The 10g Semmes Weinstein monofilament (NovoMi× 30) was used to screen diabetic patients for peripheral neuropathy. Patients was asked to close their eyes while the monofilament is pressed perpendicular 9 areas of the plantar surface and one on dorsal surface of the feet until it buckles. The patients were asked to say ‘Yes’ each time they feels the filament. Failure to feel the filament at 4 of 10 sites is 97% sensitive and 83% specific for identifying loss of protective sensation [19].

Ankle Brachial Index (ABI), the ratio of SBP of the brachial artery to that of pedal artery was measured to assess lower limb blood flow to rule out peripheral vascular disease. A bedside, Hand-held Doppler ultrasound machine (MEDA SONICS), 8 MHz probe was used, Ultrasound transducer gel, phygmomanometer and cuff, and a Calculator. Scores were: < 0.9 abnormal, 0.9 to 1.0 normal, and > 1.3 non-compressible.

Fasting blood samples for total serum cholesterol, high-density lipoproteins (good cholesterol), low-density lipoprotein (bad cholesterol), serum triglycerides and blood glucose, estimation was taken. Five milliliters of venous blood was taken from the antecubital fossa and placed in empty sterile tubes. The samples were taken to the laboratory for analysis within a day. Quality assurance prior to carrying out sample processing and analysis was adhered to. Blood samples for Glycosylated hemoglobin was collected and analyzed at the diabetic clinic, using DCA 2000+ (Bayer).

The IIEF questionnaire has 15 questions and the total score is obtained by the sum of the individual scores of each question. It addresses and quantifies five domains: erection function, orgasmic (ejaculation) function, sexual desire (libido), intercourse satisfaction (ability to sustain intercourse), and overall satisfaction/premature ejaculation domains. It classifies individual sexual function domains into mild, moderate or severe form depending on scores.

Questionnaires were checked every day after interviews for consistence filling. Then data were entered into Statistical Package for Social Studies (SPSS) version 15.0 software (SPSS, Chicago, IL, USA) which was used in the analysis of the data. Some smaller categories were aggregated into larger categories to make a statistical meaning. First descriptive statistics were calculated. Then bivariate analysis was done using Chi-square (X^2^) for categorical variables and t-test for dichotomous and continuous variables. Multivariate Logistic regression was run to identify ad quantify the adjusted odds predictors of ED. These predictors were adjusted for age, education, gender, and some other demographic variables. A 2-tailed p-value of 0.05 was taken as statistically significant.

This study was ethically approved by the Muhimbili University College of Health and Allied sciences (MUHAS) Research Ethics Committee after meeting the required ethical standards.

The principal investigator fully explained to every prospective participant the purpose of the research and was requested to participate freely. A consent form containing details of the study regarding main purpose, role of participants, and benefits associated with participating in the study (if any) was given to each prospective participant. Those who agreed to participate in the study gave a verbal consent as well as signing a written consent form written both in English and Swahili language. Swahili language is a lingua franca in Eastern Africa. Patients who did not agree to participate in the study were not denied any services and were treated accordingly.

Patients who were found to have erectile dysfunction were counseled (those who came as a couple were counseled as thus after obtaining consent), about the available treatment options and their advantages/side effects. Prescriptions were written for those who agreed to be put on treatments while maintaining confidentiality. Patients with evidence of peripheral vascular disease were planned to regularly attend the clinic every 2months for check-up. Those with hyperlipidemia were prescribed lipid-lowering drugs and the patients newly diagnosed with hypertension were managed accordingly.

## Results

Over a period of 8 months, May to December 2011, a total of 503 male diabetic patients were attended at MNH diabetic clinic where by 312 (62%) were eligible for the current study. The mean age was 51.33 (SD=15.03). Most of them were aged 45-60 years and had attained primary level of education. The details of socio-demographic are as shown on Table 1.

**Table 1:** Socio-demographic characteristics of male diabetic patients attending Diabetic clinic at MNH (N=312)

Characteristics	Frequency (N)	%
**Age group (years)**		
< 30	35	11.2
30 – 44	55	17.6
45 - 59	113	36.2
60+	109	34.9
**Marital status**		
Married	256	82.1
Single	36	11.5
Divorced	4	1.3
Cohabiting	7	2.2
Widowed	9	2.9
**Employment**		
Peasant	71	22.8
Government	38	12.2
Private sector	57	18.3
Self-employee	114	36.5
Others	32	10.2
**Level of education**		
Informal	40	12.8
Primary	152	48.7
Secondary	82	26.3
Post-secondary	38	12.2

About four-fifth of the patients (n=251) had type 2 Diabetes Mellitus (T2DM) and one fifth (n=61) had Type 1 Diabetes Mellitus (T1DM). The mean diabetic duration of this study population was 8.99 (SD = 7.62) years with a minimum of 1year and maximum of 37years.

**Figure 1:**
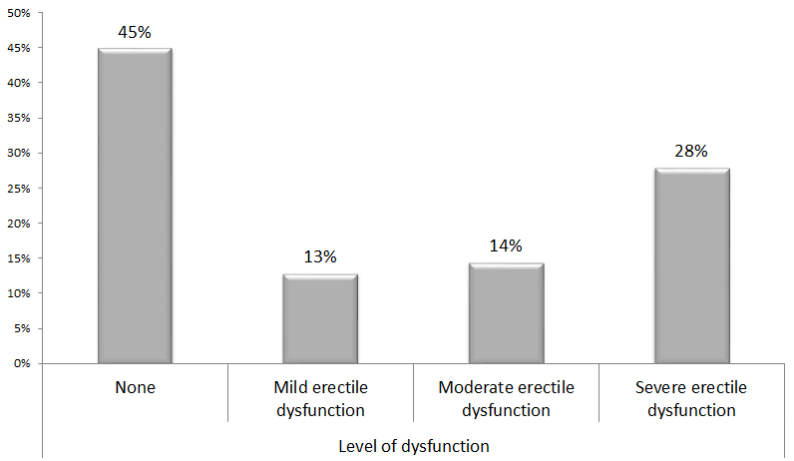
Prevalence and Severity of erectile dysfunction among male patients attending Diabetic Clinic at MNH (N=312)

**Figure 2:**
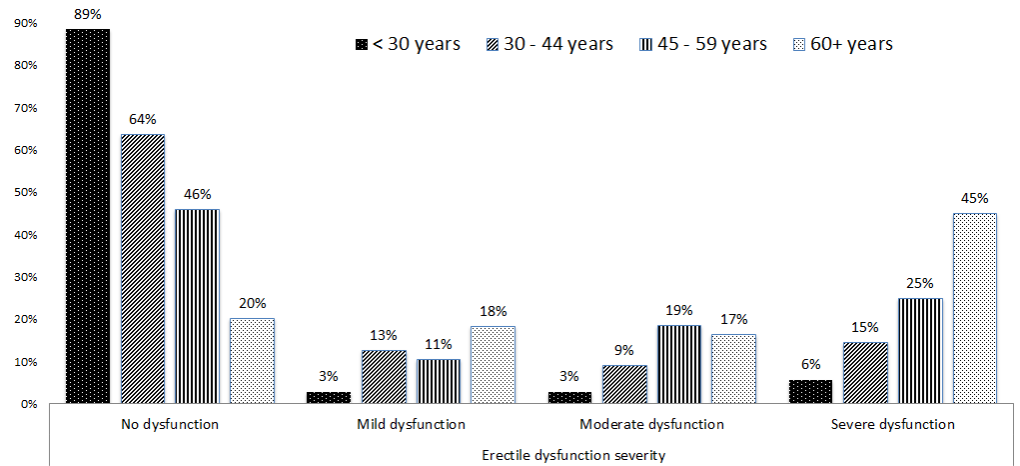
Severity of erectile dysfunction by age groups among male Diabetic patients attending Diabetic clinic at MNH in 2012 (N=312)

The prevalence of erectile dysfunction was found to be 55.1% (n=171) with the highest proportion having severe dysfunction as shown in Figure 1. As the age increases the prevalence of and severity of ED increases significantly as shown in Figure 2 below (p<0.001).

When the ED was dichotomized into “yes” and “no” options and analyzed at bivariate level there was a significant association with an increased in age, longer duration of DM (both types), and T2DM. Also ED was associated with histories of hypertension, alcohol intake and stroke as shown in Table 2. Also bivariate analysis was done to relate the prevalence of ED and clinical parameters where by only men who could not feel on monofilament test and those with abnormal ankle brachial index score were more likely to have the disorder as shown on Table 3.

**Table 2:** Bivariate analysis of socio-demographic and clinical variables associated with erectile dysfunction among male diabetic patients attending diabetic clinic at MNH

Variable	Number	Prevalence (%) of ED	p-value a
**Age group (yrs)**			
< 30	35	6 (17.1)	<0.001*
30– 44	55	23 (41.8)	
45 – 59	113	58 (51.3)	
60+	109	85 (78.0)	
**Education level**			
None	40	28 (70)	0.16
Primary	152	82 (53.9)	
Secondary	82	40 (48.8)	
Post-secondary	38	22 (57.9)	
**Current employment**			
Peasant	71	51 (71.8)	0.03*
Government	38	19 (50.0)	
Private	57	28 (49.1)	
Self-employment	114	58 (50.9)	
Others	32	16 (50.0)	
**Duration DM(years)**			
< 10	180	83 (46.1)	0.001*
10 – 19	106	70 (66.0)	
> 20	26	19 (73.1)	
**Type of diabetes**			
T1DM	61	14 (23.0)	<0.001*
T2DM	251	158 (62.9)	
**H/o smoking**			
Never smoked	252	131 (52)	0.02*
Ever smoked	60	41 (68.3)	
H/o alcohol			
Never drunk	182	92 (50.5)	0.05*
Ever drunk	130	80 (61.5)	
Hypertensive**			
No	154	68 (44.2)	<0.001*
Yes	158	104 (65.8)	
**H/o stroke**			
No	291	155 (53.3)	0.01*
Yes	21	17 (81.0)	
**H/o p/neuropathy**			
No	146	59 (40.4)	<0.001*
Yes	166	113 (68.1)	
**TOTAL**	**312**	**117 (55.1)**	

a X^2^ test

* p<0.05

** 20 patients were diagnosed with ↑BP during the study

**Table 3:** Bivariate analysis of clinical parameters associated with erectile dysfunction among male diabetic patients attending diabetic clinic at MNH (N=312)

Variable	Number	Prevalence (%) of ED	p-value a
**Monofilament test**			
Could feel	217	95 (43.8)	<0.001*
Could not feel	95	77 (81.1)	
**LDL-c (mmol/l)**			
Normal (≥3.34)	210	118 (56.2)	0.59
Abnormal (<3.34)	102	54 (52.9)	
**Triglyc (mmol/l)**			
Normal (≤ 2.3)	266	149 (56.0)	0.45
Abnormal (>2.3)	46	23 (50.0)	
**Total chol (mmol/l)**			
Normal (≤ 5.2)	209	113 (54.1)	0.59
Abnormal	103	59 (57.3)	
**Fasting b/sugar**			
Normal (≤ 7)	103	55 (53.4)	0.66
Abnormal (>7)	209	117 (56.0)	
**HbA1c (%)** b			
Normal (≤ 7%)	32	20 (62.5)	0.49
Abnormal (7%)	113	63 (55.8)	
**Ankle Brachial Index score**			
Normal	264	127 (48.1)	<0.001*
Abnormal	48	45 (93.8)	
**Body mass index** c			
<18.5	20	9 (45)	0.27
18.5 – 24.9	152	77 (50.3)	
25 – 29.9	90	56 (62.2)	
>30	38	20 (52.6)	

a Chi square test

* p<0.05

b HbA1c was done among 145 patients

c BMI of 12 individuals could not be determined because these patients each have one limb amputated and were not stable to stand for weight and height determination.

Variables that were significant at bivariate analysis were adjusted for potential confounders such as socio-demographics (age groups, education background), duration of diabetes, history of stroke and alcohol using multivariate logistic regression. Also identification and quantification potential predictors of ED were conducted. Table 4 shows that as the age increase the odds of having ED increased significantly e.g. age group 60+ years had almost 15 times higher odds compared to those Table 4.

**Table 4:** Multivariate logistic regression analysis of factors associated with erectile dysfunction among male diabetic patients attending diabetic clinic at MNH (N=312)

Variable *	aOR	95% CI	p-value
**Age group (years)**			
< 30	1		
30– 44	3.992	1.382 – 11.530	0.011
45 – 59	7.023	2.589 – 19.051	0.001
60+	15.730	5.567 – 44.447	0.001
Duration of diabetes (years)	1.038	0.999 – 1.078	0.056
**Type of diabetes**			
T1DM	1		
T2DM	2.695	0.947 – 7.666	0.063
**H/o smoking**			
Never smoked	1		
Ever smoked	1.421	0.722 – 2.799	0.309
**H/o p/neuropathy**			
No	1		
Yes	2.583	1.525 – 4.375	0.001
**Monofilament test**			
Could feel	1		
Couldn't feel	4.989	2.600 – 9.575	0.001
**ABI score**			
Normal	1		
Abnormal	11.316	3.247 – 39.443	0.001

* The reference category is first group in each variable

## Discussion

This hospital-based study confirms that the prevalence of ED is high among DM patients. It further confirms that duration of DM and age were independently correlated with the ED and its severity. As expected the history of peripheral neuropathy and abnormal ABI score were highly associated with ED. Erectile dysfunction was related to other sexual domains. Surprisingly type of diabetes, lifestyle histories and history of other conditions such as hypertension and stroke were related to ED at bivariate analyses but not on multivariate suggesting that they could have been confounded by other demographic variables.

The high prevalence of ED among DM patients is in line with several studies, which have been conducted in Italy, South America and other parts of the world. The prevalence of ED globally was found to be as low as 35% in UK, McCulloch et al [14] and in Italy, Fedele et al [15] to as high as 90% in Japan, Sasaki et al [20]. This indicates that this study incidence of 55% is with the global range as well. The prevalence is relatively lower than that of Kenyatta National Hospital which was found to be 74% [21]. The reason for the higher prevalence could be due to different in composition of study population and the methodology used.

However, the prevalence observed from current study is similar from that of the study done by Balde, NM et al [22] in Conakry (Guinea), whereby erectile dysfunction was (48%). The explanation for these large differences in the reported prevalence of ED among diabetic men could be due to differences in methodology (definitions used and scales) and population characteristics. Also inadequate linguistic validation and cultural adaptation of the IIEF may have had an influence on the prevalence reported in studies conducted in different ethnic communities.

Some studies conducted in high income countries [14, 15] reported low prevalence as compared to those done in developing world due to the fact that in developed world DM may be early detected and blood sugar may be well controlled and therefore the chronic complications are prevented i.e. differences in diabetic care or educated are more likely to deny ED.

With respect to age and peripheral neuropathy, this study confirms discoveries done by McCulloch [14] these parameters were highly associated with ED despite different analytical methods.

In contrast to the findings by Fedele et al [15] which showed that ED was more in T2DM than T1DM and in those with poor glycemic control, in this study these variables were not significant. The reason may be due to different geographical patterns and/or different statistical methods used. This entails that more studies with large sample may be needed to ascertain these factors in this setting. Also the findings in this study were in odds with those revealed by Khatib et al [23] which showed that apart from age other independent risk factors of ED were hypertension and poor glycemic control.

## Conclusion

This study showed that the prevalence of erectile dysfunction is high among DM patients. The study further showed that old age, peripheral neuropathy and evidence of peripheral arterial disease are main factors strongly associated with ED. These findings reinforce the need to screen for ED in diabetic patients as it is done for other chronic complications of diabetes in routine clinical practices for early detection, treatment and possibly prevention. The screening for presence or absence of ED can be best done by the use of IIEF questionnaire. Interventions aimed at prevention of DM especially type 2; early diagnosis of DM and detection of its complications e.g ED, and adherence to treatment to prevent these complication should be implemented. ED is a neglected medical problem that necessitates screening of all DM patients at each visit to aid the delivery appropriate treatment.
